# SciData: a data model and ontology for semantic representation of scientific data

**DOI:** 10.1186/s13321-016-0168-9

**Published:** 2016-10-14

**Authors:** Stuart J. Chalk

**Affiliations:** Department of Chemistry, University of North Florida, Jacksonville, FL 32224 USA

**Keywords:** Science data, Semantic annotation, Ontology, JSON-LD, RDF, Scientific data model

## Abstract

With the move toward global, Internet enabled science there is an inherent need to capture, store, aggregate and search scientific data across a large corpus of heterogeneous data silos. As a result, standards development is needed to create an infrastructure capable of representing the diverse nature of scientific data. This paper describes a fundamental data model for scientific data that can be applied to data currently stored in any format, and an associated ontology that affords semantic representation of the structure of scientific data (and its metadata), upon which discipline specific semantics can be applied. Application of this data model to experimental and computational chemistry data are presented, implemented using JavaScript Object Notation for Linked Data. Full examples are available at the project website (Chalk in SciData: a scientific data model. http://stuchalk.github.io/scidata/, 2016).

## Background

For almost 40 years, scientists have been storing scientific data on computers. With the advent of the Internet, research data could be shared between scientists, first via email and later using web pages, FTP sites, and online databases. With the advancement of Internet technologies and online and local storage capabilities, the options for collecting and stored scientific information have become unlimited.

Yet, with all these advancements science faces an increasingly important issue of interoperability. Data are commonly stored in different formats, organized in different ways, and available via different tools/services severely impacting curation [2]. In addition, data is often without context (no metadata describing it), and if there is metadata it is minimal and often not based on standards. Though the Internet has promoted the creation of open standards in many areas, scientific data has, in a sense, been left behind because of its inherent complexity. The strange part about this scenario is that scientific data itself is not the biggest problem. The problem is the contextualization of the scientific data—the metadata that describes system that it applies to, the way it was investigated, the scientists that determined it, and the quality of the measurements.

So, what is scientific data and where is the metadata? Peter Murray-Rust grappled with these questions in 2010 and concluded that it is “factual data that shows up in research papers” [3]. When writing scientific articles, researchers add most (in most cases not all) of the valuable metadata in the description of the research they have performed. The motivation of course is open sharing of knowledge for the advancement of science, with appropriate attribution and provenance of research work. As we move toward the fourth paradigm [4], where large aggregations of data are the key to discovery, it is imperative that the context of the data are articulated completely (or as completely as possible), not only to identify it’s origin and authenticity, but more importantly to allow the data to be located correctly on the “scientific data map”.

To address these issue, this paper describes a generic scientific data model (SDM)/framework for scientific data derived from (1) the common structure of scientific articles, (2) the needs of electronic notebooks to capture scientific research data and metadata, and (3) the clear need to organize scientific data and its contextual descriptors (metadata). The SDM is intended to be data format/software agnostic and extremely flexible, so that it can be implemented as the scientific research dictates. While the SDM is abstract in nature, it defines a concrete framework that can be easily implemented in any database and does not constrain the data and metadata that can be stored. It therefore serves as a backbone upon which data and its associated metadata can be ‘attached’.

In addition, this paper describes an ontology that defines the terms in the SDM, which can be used to semantically annotate the structure of the data reported. In this way, scientific data can be integrated together by storage in Resource Description Framework (RDF) [5] triple stores and searched using SPARQL Protocol and RDF Query Language (SPARQL) queries [6].

The use of the ontology in the generation of RDF is demonstrated in examples of scientific data saved in JavaScript Object Notation (JSON) for Linked Data (JSON-LD) [7] format using the framework described by the SDM. From these examples it is shown how useful a hybrid structured (relational)/graph (unstructured) approach is to the representation of scientific data.

JSON-LD is a recent solution to allow transfer of any type of data via the web’s architecture—Representational State Transfer (REST) [8]—using a simple text-based format—JSON. [9] JSON-LD allows data to be transmitted with meaning, that is, the “@context” section of a JSON-LD document is used to provide aliases to the names of data reported and link them to ontological definitions using a Uniform Resource Identifier (URI)—often a Uniform Resource Locator (URL). In addition, the structure/data of the JSON-LD file can be automatically be serialized to Resource Description Format (RDF) using a JSON-LD processor, e.g. the JSON-LD Playground [10]. This capability makes JSON-LD files not only useful as a data format but also a compact representation of the meaning of the data.

## Methods

### Aim, design and setting of the study

The aim of this work was to develop a serialization of scientific data and its contextual metadata. The design was encoded using the JSON-LD specification [7] because it is both a human readable and editable format and can easily be converted to RDF [5] triples for ingestion into a triple store and subsequent SPARQL searching [6]. The intent was that the data model, developed to afford the serialization, would be able to structure any scientific data (see examples).

### Description of materials

Data were taken from different data sources and encoded in the proposed serialization. Items 5, 6, and 7 were created using XSLT files.

1. laboratory notebook data
2. research article data
3. spectral data (NMR)
4. computational chemistry data
5. PubChem download as XML
6. Dortmund Data Bank webpage as HTML
7. Crystallographic Information Framework (CIF) file as text

### Description of all processes and methodologies employed

In this work different pieces of scientific data were selected and an analysis performed of the required metadata that was necessary to completely describe the context of how the data were obtained. After looking at the data and its context, reading a number of research articles on what scientific data is, and reviewing journal guidelines for submission of research, a preliminary generic structure of scientific data and metadata was developed. This was iteratively improved by encoding the data of higher and higher complexity into the framework and adding/deleting/adjusting as necessary to make the model fit the needs of the data.

### Statistical analysis

Statistical analyses were not performed.

## Results and discussion

### Considerations for a scientific data model

#### What is scientific data?

In order to appreciate what scientific data is we took a step back and looked at the scientific process to abstract the important aspects that underpin the framework of what scientists do and how they do it. When we teach students to think and act like scientists we start with the general scientific method [11]:

- *Define a research question* What is the scope of the work? What area of science is the investigation in? What phenomena are we investigating?
- *Formulate a hypothesis* What parameters/conditions do we control or monitor in order to evaluate the effect on our system?
- *Design experiments* What instrumentation/equipment do we use? What are the settings and/or conditions? What procedures are used?
- *Make observations* What are the values of the controlled parameters, experimental variables, measured data, and/or observations?
- *Generate results* How is data aggregated? What calculations are used? What statistical analysis is done?
- *Make conclusions/decisions* What are the outcomes? Is the data good quality? Do they help answer the question(s) asked? How does the data influence/impact subsequent experiments?

The process above defines the types of information scientists collect as they perform science and once a project is complete they aggregate all of the important details (data, metadata, and results) from the process and synthesize one or more research papers to inform the world of their work. Thus, scientific papers can be considered a pseudo data model for science. Yet, this format has significant flaws as, in general, it is not typically setup uniformly, often has only a subset of all the metadata of the research process, and is influenced by the biases of authors and the constraints of publication guidelines.

#### How is scientific data structured?

Scientists have grappled with structuring scientific data since its inception. Communication of scientific information in easy to understand formats is extremely important for comprehension and hypothesis development, especially as the size and complexity of data grows. Its representation is also highly dependent on the research area both in terms of size/complexity of captured data and common practices of the discipline.

In chemistry the best example of data representation is the periodic table [12], the fundamental organization of data about elemental properties, structure and reactivity, and it is impossible to be chemist without appreciating the depth of knowledge it represents. The same is true in biology about the classification of species [13, 14], or in physics the data model underlying the grand unification of forces [15].

Data representation/standardization in chemistry has since evolved primarily in two areas: Chemical structure representation and analytical instrument data capture [16].

## Chemical structure representation

Communication of chemical structure has been an area of significant development since John Dalton introduced the idea that matter was composed of atoms in 1808, and developed circular symbols to represent known atoms [17]. It wasn’t long before Berzelius wrote the first text based chemical formula, H_2_SO_4_, showing the relative number of atoms of each element. Since these early steps chemists have found need to create representations of molecular structure for many different applications. In the Twentieth century this has brought us text string notations such as Wiswesser Line Notation (WLN) [18], simplified molecular-input line-entry system (SMILES) [19], and most recently the International Chemical Identifier (InChI) [20] in addition to the classical condensed molecular formula. Both SMILES and InChI are elegant solutions to encoding structural information in text where the string to structure conversion (and vice versa) can be done accurately by computer for small molecules. Solutions for large molecules, crystals and polymers are still needed, as are definitive representation of stereocenters.

**Fig. 1:**
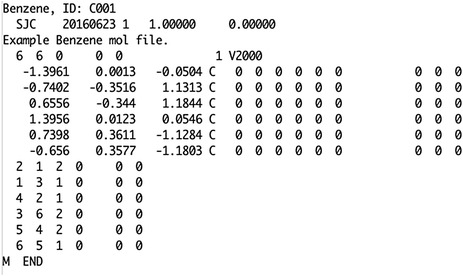
Example MOL file format for benzene

Chemical structure representation on computers, using standard file formats, has been a challenge many have attempted to solve. Currently, there are over 40 different file formats (see [21]) for 2D, 3D, and reaction representation. Of these, the.mol file (MOL) V2000 [22] is the most widely available even though the V3000 format has been out for many years. The MOL file, like many others contains a connection table that defines the positions of, and bonds between, the atoms (Fig. 1).

**Fig. 2:**
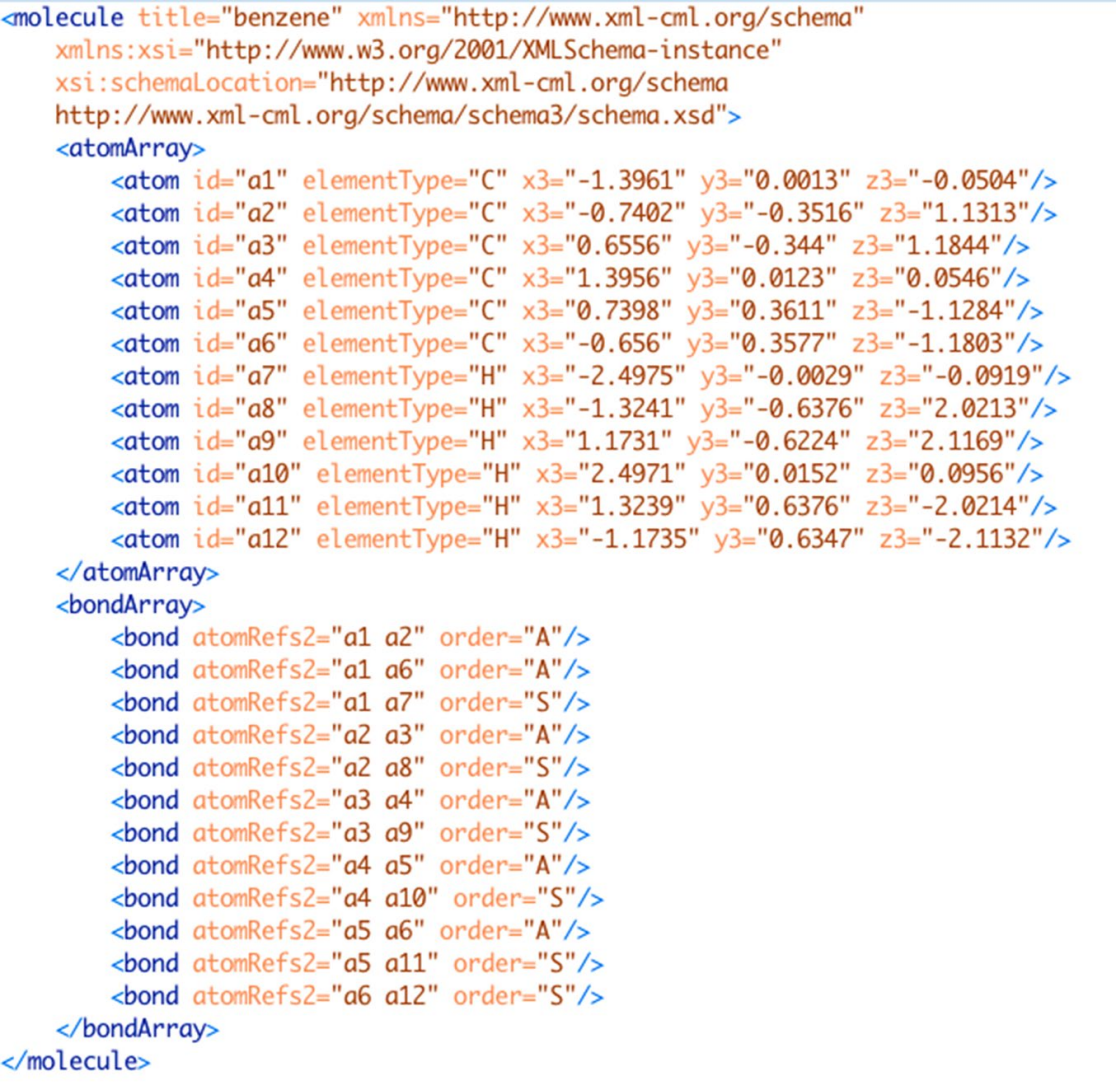
Example CML file format for benzene

In addition to MOL files, the Chemical Markup Language (CML) [23], an Extensible Markup Language (XML) [24] format, is a more recent development allows the content and structure of the file (through use of an XML schema) to be validated. This is an important feature for reliable storage and transmission of chemical structural information and provides a mechanism, through digital signatures, to ensure integrity of the files. Figure 2 shows the equivalent, valid CML file for the MOL file in Fig. 1. While the CML is larger (1931 vs. 721 bytes) it is easier to read by humans (and computers) and contains information about the hydrogen atoms where the MOL file does not.

**Fig. 3:**
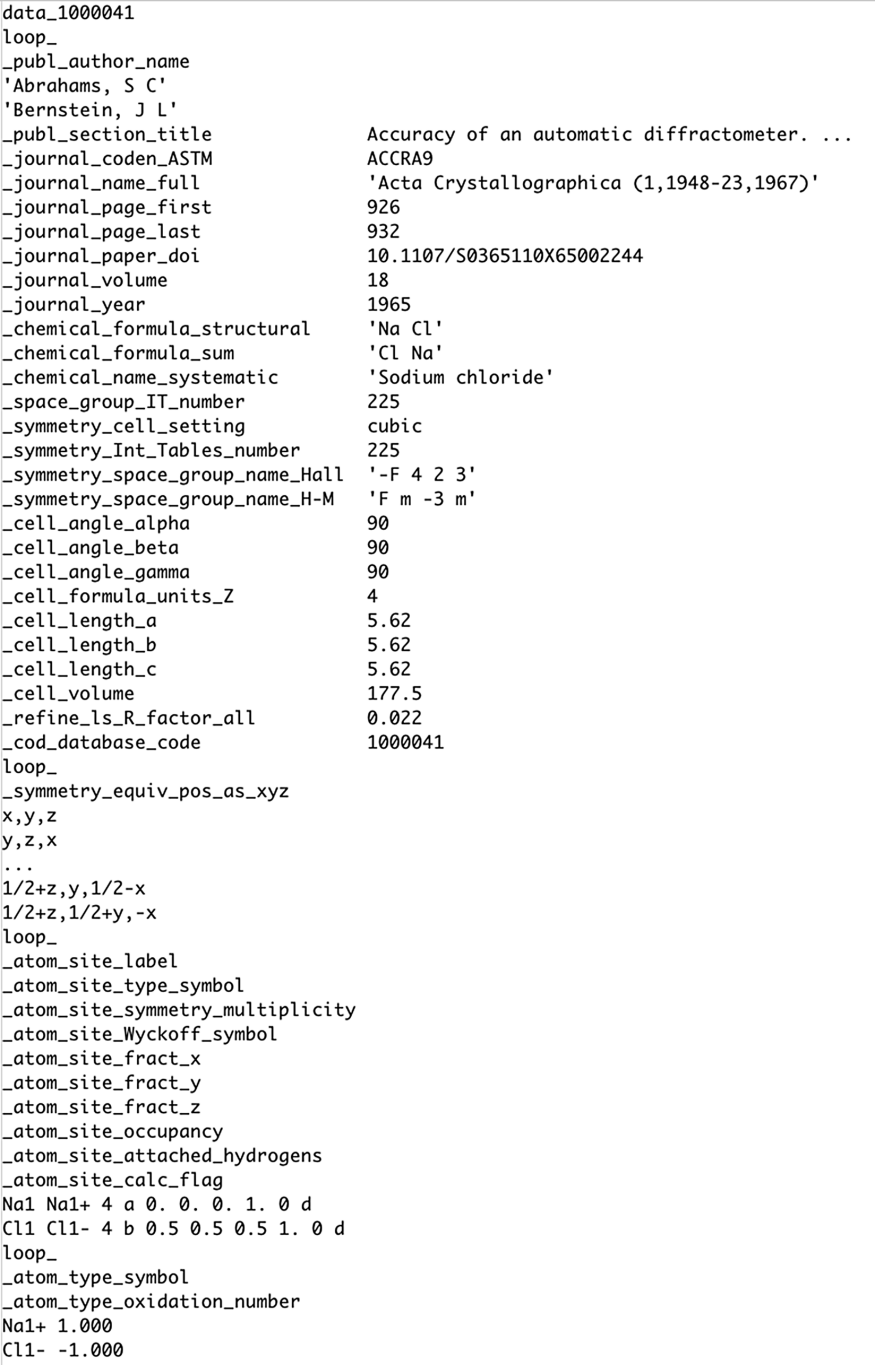
Example CIF file for NaCl

Finally, the exemplar chemical structure representation standard for data reporting is the Crystallographic Information Framework (CIF) developed in 1991 [25–27] as an implementation of the Self-defining Text Archive and Retrieval (STAR) format [28]. The CIF/STAR format uses a similar approach to JCAMP-DX (see below) in that a number of text strings are defined to identify specific metadata/data items. The use of well-defined labels is not only more extensive in CIF but the format also includes the option to create pseudo tables of any size using the loop_ instruction, whereas JCAMP is limited to two columns (XY data or peak tables). The format has evolved significantly from its inception due to community input and support and is now integrated into the publishing of crystallographic data in journal articles through the Cambridge Crystallographic Data Centre (CCDC). Figure 3 shows an example CIF file for NaCl.

### Analytical instrument data capture

Since the introduction of microcomputers in the early 1970’s, chemists have used a number of formats to deal with the large amounts of data produce by scientific instruments. The significant initial limitation, that of available storage space, resulted in two different approaches (1) the use of a ASCII text file format (JCAMP-DX) [29] with options for text based compression of data and (2) binary file format (netCDF) [30] where the file structure is inherently more space efficient. Both the Analytical Data Interchange (ANDI) format [31, 32] (built using netCDF) and JCAMP-DX are still in use today with the JCAMP-DX specification more prevalent because of its text-based format.

**Fig. 4:**
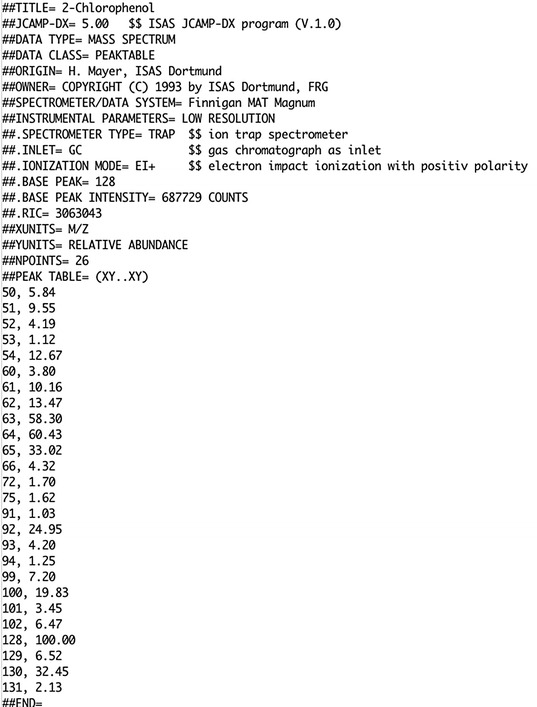
JCAMP-DX format mass spectrum file for 2 chlorophenol

The Joint Committee on Atomic and Molecular Physical Data (JCAMP) under the International Union of Pure and Applied Chemistry (IUPAC) has published a number of versions of the data exchange (DX) standard for near-infrared, infrared, and ultraviolet–visible spectrophotometry, mass spectrometry, and nuclear magnetic resonance. JCAMP-DX is a file specification consisting of a number of LABELLED-DATA-RECORDs or LDRs. These are defined to allow reporting of spectral metadata and raw/processed instrument data. Figure 4 shows an example mass spectrum in JCAMP-DX format.

Although the JCAMP-DX file format is widely used for export and sharing of spectral data, the specification has not been updated for over 10 years and as a result has limitations in terms general metadata support (static set of LDRs), technique coverage, and is prone to errors/alteration for unintended uses—which breaks compatibility with readers. As a result, an effort was started in 2001 to develop an XML format to replace the suite of JCAMP-DX specifications. The Analytical Information Markup Language (AnIML) [33] is an effort to ‘develop a data standard that can be used to store data from any analytical instrument’. This lofty goal has led to a long development process that will be completed in 2016, and result in a formal standard through the American Society for Testing and Materials (ASTM).

**Fig. 5:**
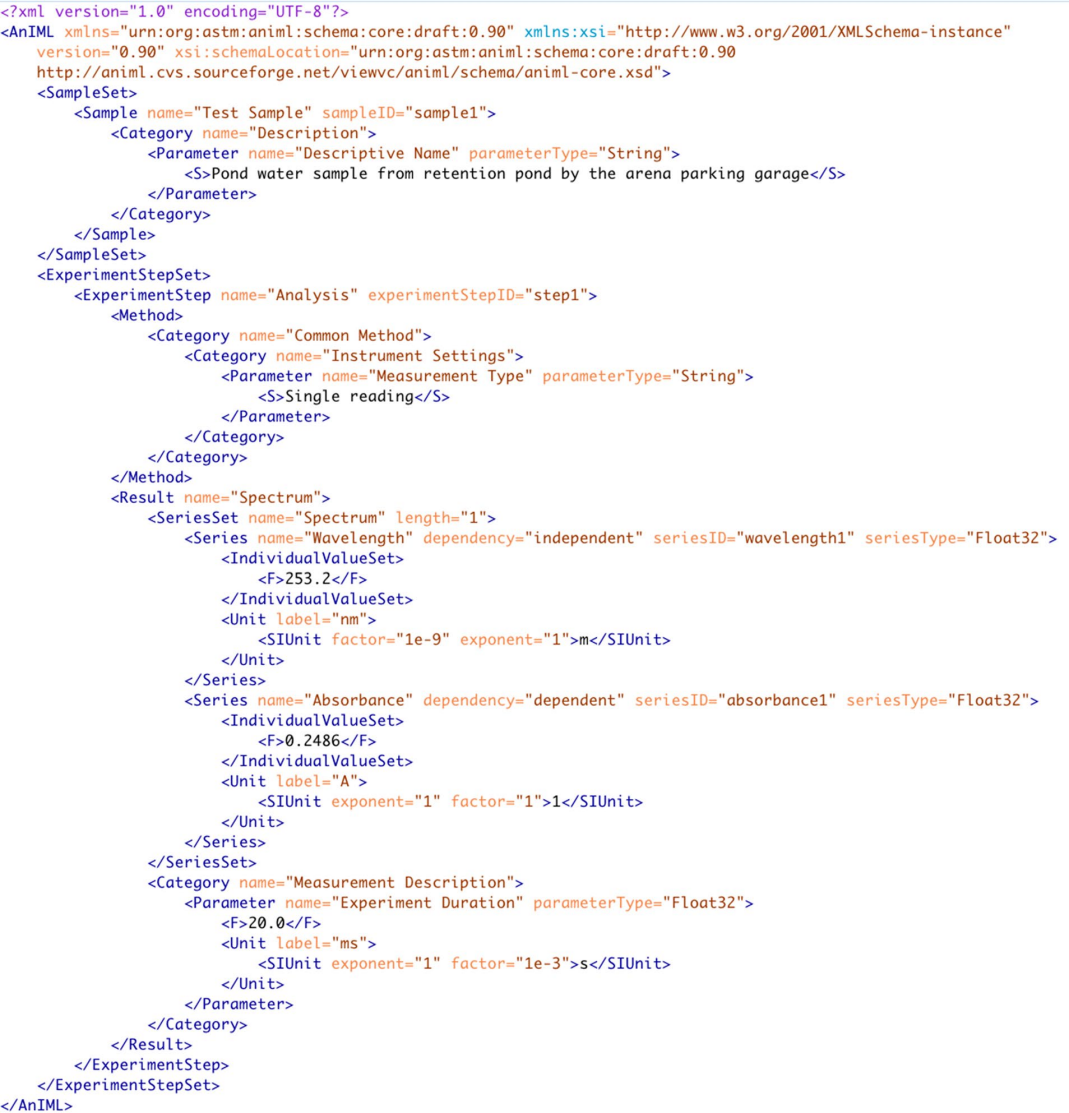
Example AnIML file—a single reading of absorbance

AnIML defines a core XML schema for basic elements that will contain data and then uses an additional metadata dictionary, and AnIML Technique Definition Document (ATDD) to prescribe the content of an AnIML file for a particular instrumental technique [33]. This approach makes the format flexible so that it can be used to represent data of all types, from a single datapoint, to a complex array of three-dimensional data. In addition, information about samples, sample location (relative to introduction into an instrument), analytes and instrumental parameters are stored with the raw instrument data. Figure 5 shows an example AnIML file.

#### How is scientific data stored?

In addition to knowing what scientific data is and how it is represented, it is important to consider how it is stored (and hopefully annotated). Outside of scientific articles, scientific data is published in many databases where the data can be compared with other like data in order to show trends/patterns and afford a higher-level of knowledge mining. Commonly, these are implemented using Structured Query Language (SQL) based relational databases such as MySQL [34], MS SQL Server [35], or Oracle [36]. These software store data in tables and link them together via fields that are unique keys. SQL based software is very good for well-structured information that can be represented in a tree format (rigid schema). However, large sets of research data do not fit rigid data models, as by its very nature scientific data is high variable in structure.

Advances in the area of big data have attempted to address the non-uniformity in aggregate datasets by using different data models. Recently, there has been a major shift toward graph databases in support of big data applications across a variety of disciplines. Storing and searching large, often heterogeneous, datasets in relational databases creates problems with speed and scale up [37]. As a result, many companies with large amounts of data have turned to graph databases (one of many NoSQL type databases where ‘NoSQL’ stands for ‘Not only SQL’) where data is stored as RDF subject-object-predicate ‘triples’. In comparison to relational databases, graph databases are considered schema-less where the organization of the data is more natural and not defined by a rigid data model. Essentially, any set of RDF subject-predicate-object triples can be thought of as a three-column table in a relational database. Software used to store RDF data is called triple stores [38]—or quad stores [39] if an additional column for a named graph identifier is added. Data in these databases can be searched using the World Wide Web consortium (W3C) defined SPARQL query language [6].

In chemistry there are many websites that show the power of using a database to store large amounts chemical data made available for free or via paid access. Increasingly these sites are being used for basic research and industrial applications as they provide a way to; identify property trends; search for the existence of compounds; show property-structure relationships; and create datasets to build system models. Some highlights are:

- PubChem [40]—chemical, substance, and assay data available with over 91 million compounds. Has user API to downloading data and RDF querying.
- ChemSpider [41]—chemicals, instrument data, and property data for over 56 million compounds. Links to suppliers, literature articles, patents. Has limited API and RDF/XML download
- Dortmund Data Bank [42]—curated property data for over 53,000 compounds. Limited set can be searched for free.
- Cambridge Crystallographic Data Centre [43]—over 833,000 crystal structures (CIF files). Limited set can be searched for free.

#### What is the best way to communicate context?

Given that the global aggregation of research data is the goal, an important component that is needed relative to any type of framework is a formal definition of the meaning of the data and metadata (contextual data). As mentioned above, current scientific practices are lacking in the generation/reporting of contextual data as researchers are only considering their audience to be human (where meaning is either implicit or can be inferred). If data/metadata is migrated to computers systems, some mechanism to articulate the meaning of the data and metadata is required as storing text in a database is just that—text—to a computer. Through the development of the semantic web this can be achieved through the use of an ontology, or a suite of ontologies. Ontologies are the ‘formal explicit description of concepts in a domain of discourse’ [44], or an agreed standard for describing the concepts within a field of study. In the recent move toward the semantic web, the importance of ontologies and their unified representation cannot be understated. In 2004 (and updated in 2009) the W3C released the Web Ontology Language (OWL) [45] as a standard way to represent ontologies in RDF.

#### How best to save, organize, archive, and share data?

Even with all the developments mentioned above there are still challenges that have not been solved. In a nutshell, the problem is that the solutions currently available have been built in isolation (by necessity limiting the scope makes projects more tractable), have little/no machine actionable semantic meaning, are too rigid, are not easy to extend (without breaking existing systems), and are tied heavily to their implementation. As a result, although data is available from many sources it is difficult and time consuming to integrate that data. It is also difficult to search across this heterogeneous pool of information as everyone identifies things differently—there is no broad use of agreed ontological definitions of terms.

A solution to these problem requires abstracting the scenario to a higher level where the structure of the data is normalized in the broadest sense such that any data/metadata can be placed in that structure. This is the essence of the SDM. It does not try to define the data/metadata needed to accurately record and contextualize the scientific data, rather it defines its metaframework, and via an ontology its meaning.

**Fig. 6:**
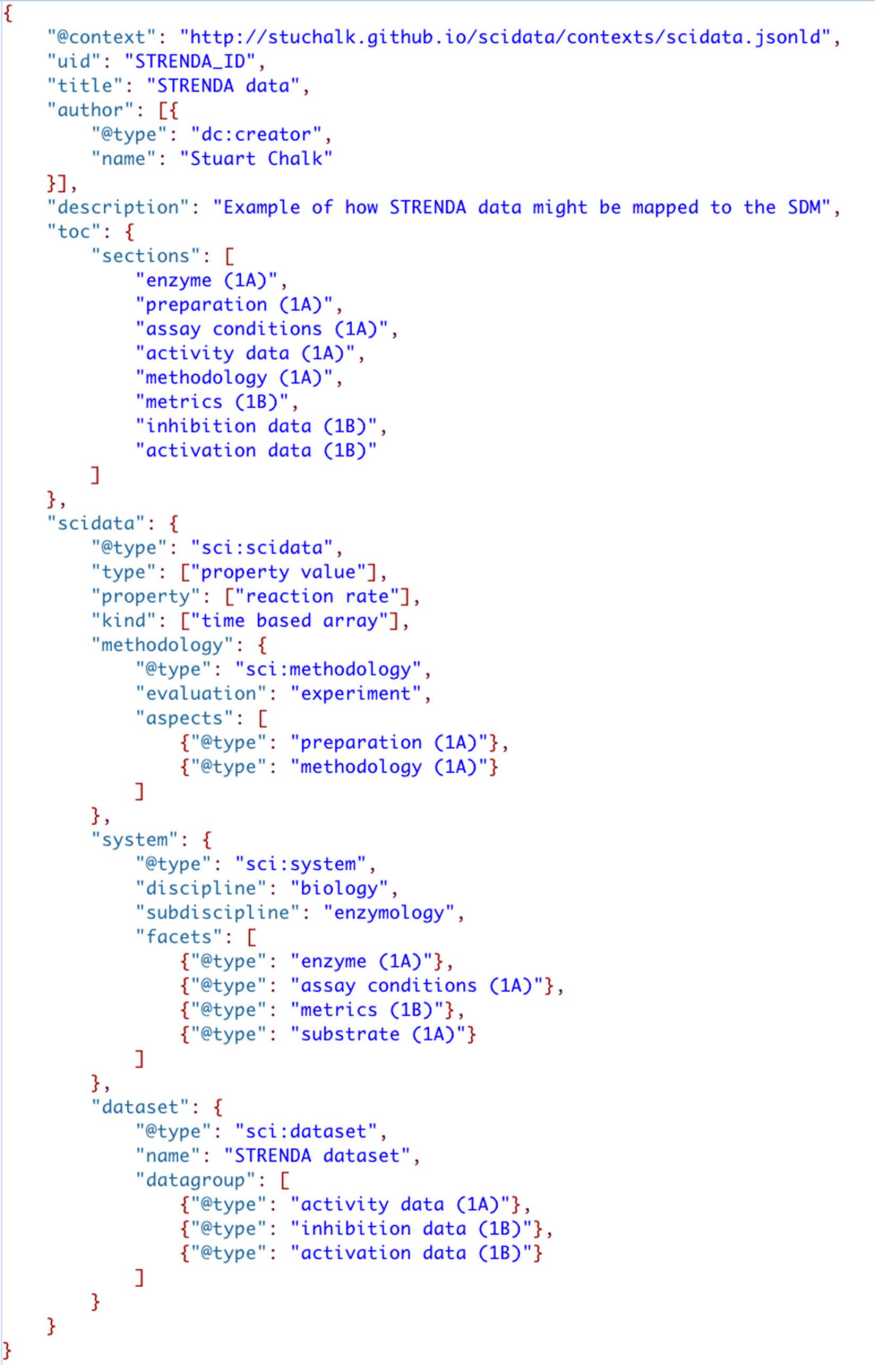
STRENDA Data Categories [52] mapped into the SDM structure

The task of defining the meaning of data and metadata that is placed in any metaframework is the purview of the discipline, where standard ontologies should be developed/refined and implemented. Although this might seem a significant challenge, previous work to standardize the reporting of chemical data can be repurposed to fit this need. For instance, metadata on safety would logically come the new Globally Harmonizes System (GHS) of Classification and Labeling [46], metadata for functional groups of organic compounds would come from the IUPAC Blue book on organic compound nomenclature [47], or for inorganic naming from the IUPAC Red Book [48]. In the biosciences existing work on ‘minimal information standards’ such as the Minimal Information About a Microarray Experiment (MIAME) [49], Minimal Information Required for a Glycomics Experiment (MIRAGE) [50], and Standards for Reporting Enzymology Data (STRENDA) [51] could be reused in the SDM without much alteration. Figure 6 shows an example of how categories of STRENDA data/metadata could logically be mapped to the SDM.

In order to reinvent how science saves, searches, and re-uses data the implemented solution must have a low barrier to adoption by scientists. While the individual researcher may be excited to use a globally searchable dataset(s), they do not want to be burdened with IT related issues in order to access or implement it. Although the SDM is designed to be format/implementation agnostic, the JSON-LD standard is perfect for representation of the data model as it is a simple text-based encoding, that can handle the types of data needed for the model, and is built to translate to RDF. Examples below that use the SDM are formatted in JSON-LD.

The goal of science is to share research data such that the community can search and use it to advance science. Based on the discussion above, initially one might think that a system for this should be based on a graph database because of its inherent flexibility (anything can be linked to anything) as opposed to relational databases (where data is in tables and linked via unique keys). However, implementing a graph database without any kind of structure would be equivalent to trying to search the current heterogeneous landscape of research data—impossible because nothing is standardized (for example, think about how many ways a scientist could indicate that they used spectrophotometry in their work). What is needed is a hybrid model where a framework for the data and metadata from scientific experiments is used to provide organization (separate from the scientific data/metadata), yet allows flexibility in the types of data put on the framework via creation of discipline specific descriptions and/or ontologies. This is the premise behind the development of the SDM.

### Description of the SciData scientific data model

Detailed below is an initial attempt to create a framework upon which to organize scientific data and its metadata. It is by no means a definitive or complete framework and serves only as a starting point to demonstrate the potential of this idea, and act as a catalyst to encourage other scientists to contribute to its development. None of the elements described below are required, other elements can be added (as long as they have a semantic definition and logically fit the scope), and all elements are open to revision (readers are encouraged to provide feedback). Readers are also encouraged to visit the project website [1] for the current version of the data model.

**Fig. 7:**
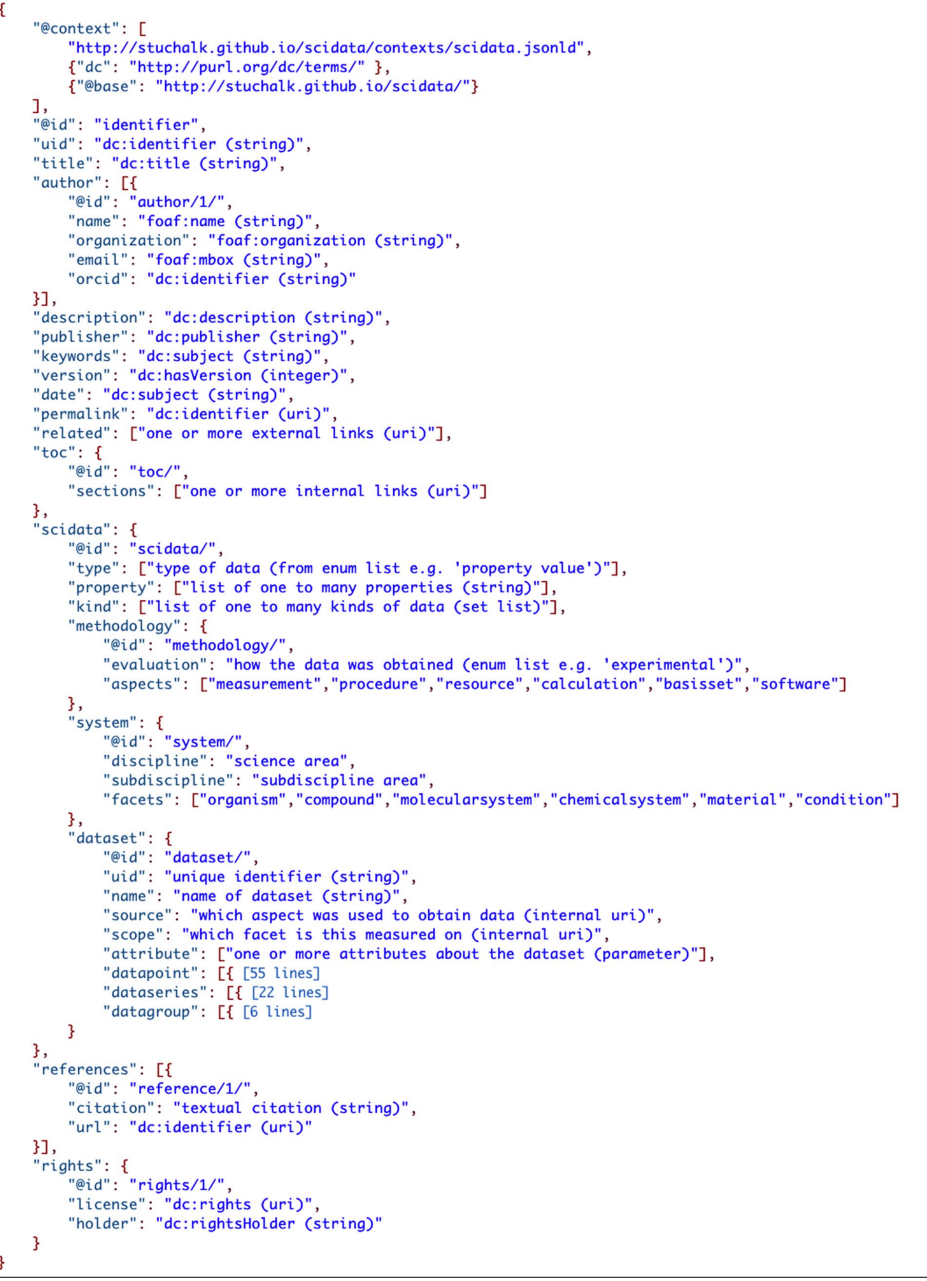
The top-level structure of the SciData Data Model (information in [] indicates the number of lines of hidden code, “dc” stands for “Dublin Core”)

Figure 7 shows a JSON-LD file that outlines the data model framework. The root level of the structure (everything other than ‘scidata’) contains general metadata to describe the “data packet”, i.e. attribution and provenance. The ‘toc’ attribute is use to articulate the kinds of methodology ‘aspects’, system ‘facets’, and ‘dataset’ elements the report contains. This is an important feature relative to the federated search of data as mechanisms to limit the size/scope of searches will be important if a global search of such data is to be realized.

The generic container for the data and metadata in the model is ‘scidata’. This contains metadata descriptors for the types and formats of data, as well a list of the properties for the data that is being reported. What follows are the three main sections that describe the research undertaken: ‘methodology’, ‘system’, and ‘dataset’.

#### Methodology

 Similar to the ‘Experimental’ section in a research paper the ‘methodology’ section contains metadata about how laboratory experiments, computer calculations, or theoretical analysis was done to arrive at the data in the packet. The ‘evaluation’ term indicates which of these approaches was used to obtain the data. Each of the different ‘aspects’ of the methodology is reported as a separate section (JSON object) and any/all metadata that are relevant to the methodology of the research can and should be included. Although in the diagram above the aspects of ‘measurement’, ‘procedure’, ‘resource’, ‘calculation’, ‘basisset’, and ‘software’ are shown, these are just examples of aspects that might be reported here. The SDM defines only ‘methodology’, ‘evaluation’ and ‘aspects’ here with metadata under ‘aspects’ included as needed/available, be it semantically annotated or not. It is envisioned that both general and discipline specific ‘aspects’ will be developed based on domain specific agreement on best practices for inclusion of minimal metadata and/or default ontological definitions (as discussed above).

#### System

 The ‘system‘section contains data that is normally reported in different places in a research article. For any scientific research that is performed there is a system that the research is working with/on. A description of the compound(s), organism(s), or material(s) that the data is about needs to be articulated so that the scope of what the data describes can be characterized. It is also important to be able to report the condition(s) under which the data was recorded, the time-point or timeframe at or over which it was performed, etc. Several example facets are listed in the schema below, but again none are required and others can be included as needed to characterize the data. Just like the ‘methodology’ sections’ ‘aspects’, the ‘system’ sections’ ‘facets’ is a flexible part of the framework that can hold metadata about one to many ‘facets’ in addition to general descriptive terms about the discipline that the data is nominally from. Again the data model defines only ‘system’, ‘discipline’, ‘subdiscipline’, and ‘facets’ here with metadata under ‘facets’ included as needed/available, be it semantically annotated or not.

#### Dataset

**Fig. 8:**
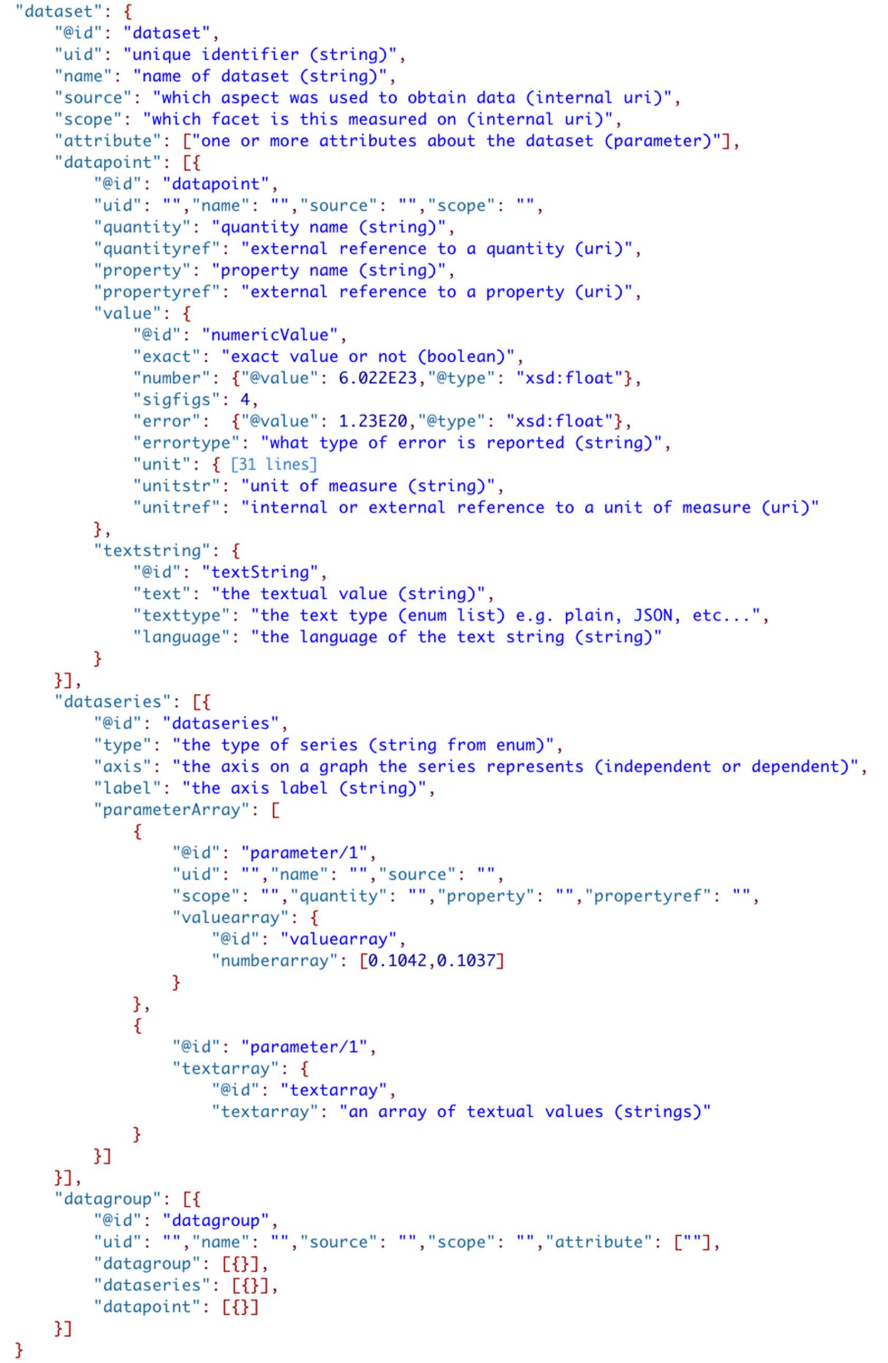
The dataset structure of the SciData Data Model

 The final section of ‘scidata’, the ‘dataset’, is of course the most important (Fig. 8). Dataset is used as a descriptor here to indicate that it is a generic container for data that can logically be reported as a set. The level and scope of the aggregation for a ‘dataset’ can be at any scale (and is at the discretion of the researcher) and thus it can be used to report a single piece of data or all of the data from a large research study. Within ‘dataset’ data can be organized/reported in multiple ways. Individual pieces of data are added to the ‘datapoint’ section and it is implied that there is no relationship between values included. Data that is logically related to other data, either as a time or property series or correlated data such as a spectrum (multiple correlated arrays) are stored in the ‘dataseries’ section, either directly under ‘dataset’ or as part of a ‘datagroup’. Here the array of data that is recorded can be reported as a JSON array, or as a JSON array of internal links (IRI’s) to ‘datapoint’ data. This allows logical arrays of data to be efficiently stored while also allowing series of datapoints that are collected at different times to also be represented.

A ‘datagroup’ section is used where there is a need to aggregate data together based on a higher-level structure, and the ability to nest a ‘datagroup’ inside another ‘datagroup’ makes for hierarchical organization of data that fits the researcher need. It is also important to point out here that the use of ‘datagroup’, ‘dataseries’, and ‘datapoint’ on their own do not provide a semantic meaning of how the data relates to anything else, rather they are a way to compartmentalize data so that it can be related to other things through the use of the ‘source’ (methodology ‘aspect’) and ‘scope’ (system ‘facet’) metadata elements. In reports that contain large datasets, across many experiments, this structure provides the maximum flexibility to report data yet still affords the structure that the data model provides.

Also, in the dataset sub-framework there are references to ‘parameter’ types for certain elements. The ‘parameter’ object is a generic structure that is used as the basis for metadata in ‘datapoints’, ‘attributes’, ‘conditions’ (under ‘system’), ‘settings’ (within ‘methodology’) and many more. A parameter is a report of a property (with/without its quantity) and its value and thus can be used to describe a wide variety of data/metadata in the data model. Parameter values may either be numeric (‘value’) or textual (‘textstring’), single values or arrays of values. Numeric values are described by metadata that indicates its type (decimal, integer, float, etc.), significant figures, error, and if the value is an exact number or not (useful for calculations). Text values are described by their type (plain, JSON, Extensible Markup Language (XML), etc.) and language. Implementers of the SDM can use the ‘parameter’ type in the definition of ‘aspects’ or ‘facets’ instead of having to invent their own data structures. This makes implementation easier and more consist and other parts of the SDM can be re-used in the same manner.

**Fig. 9:**
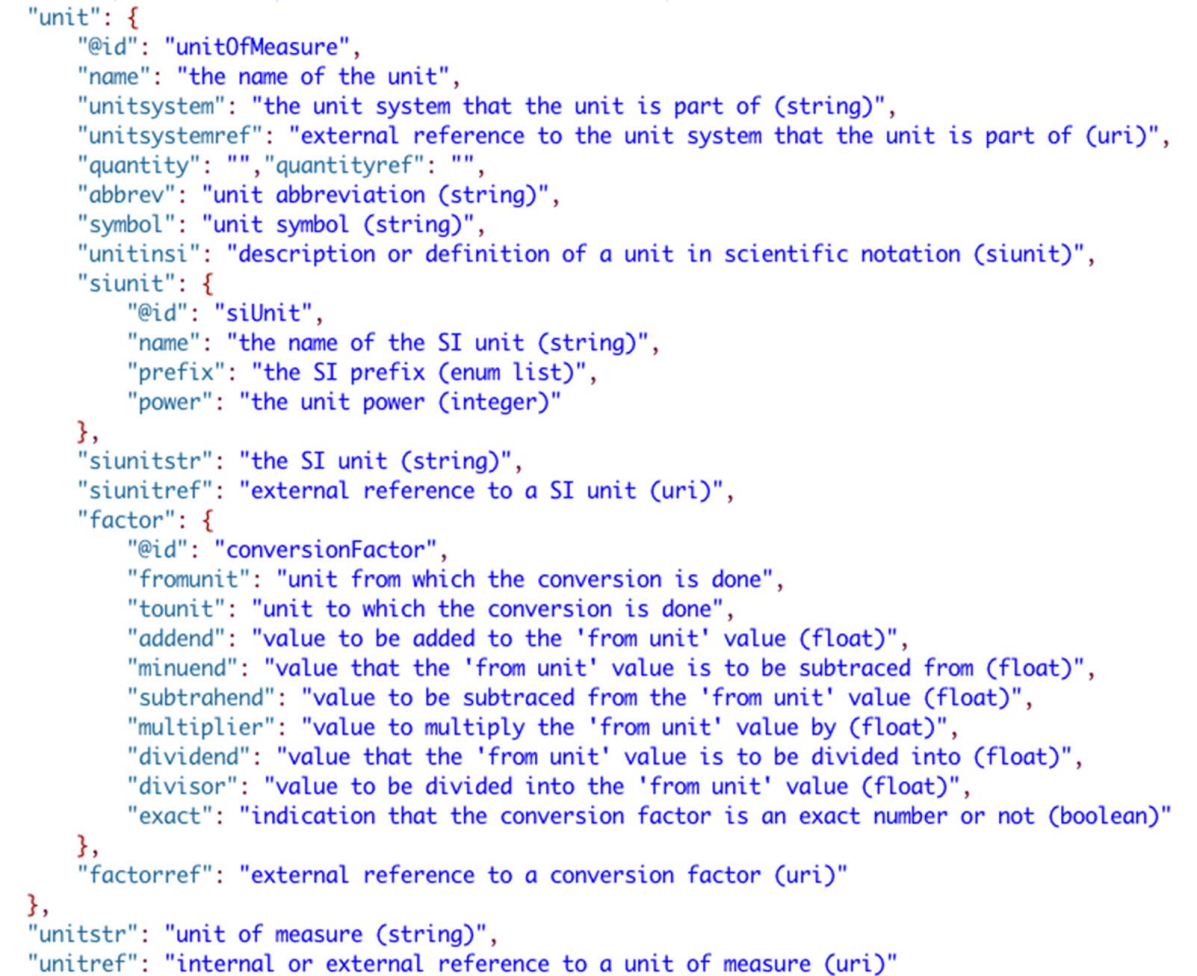
Metadata for units in the SciData data model

The final, and most fundamental piece of the data model is the representation of units of measure (Fig. 9). Unit metadata is designed to accurately represent any unit likely to be used in the context of scientific research as well as reference other representations of units (via ‘unitref’). The user can report a unit without defining it (using ‘unitstr’), define it in place using the metadata shown in Fig. 9 (‘unit’), or reference a unit defined elsewhere (internally or externally) in the report via ‘unitref’. The specification has been written to integrate online representations of units, quantities, prefixes and unit conversion factors, such as those currently available in the QUDT ontologies [53].

### A scientific data model ontology

**Fig. 10:**
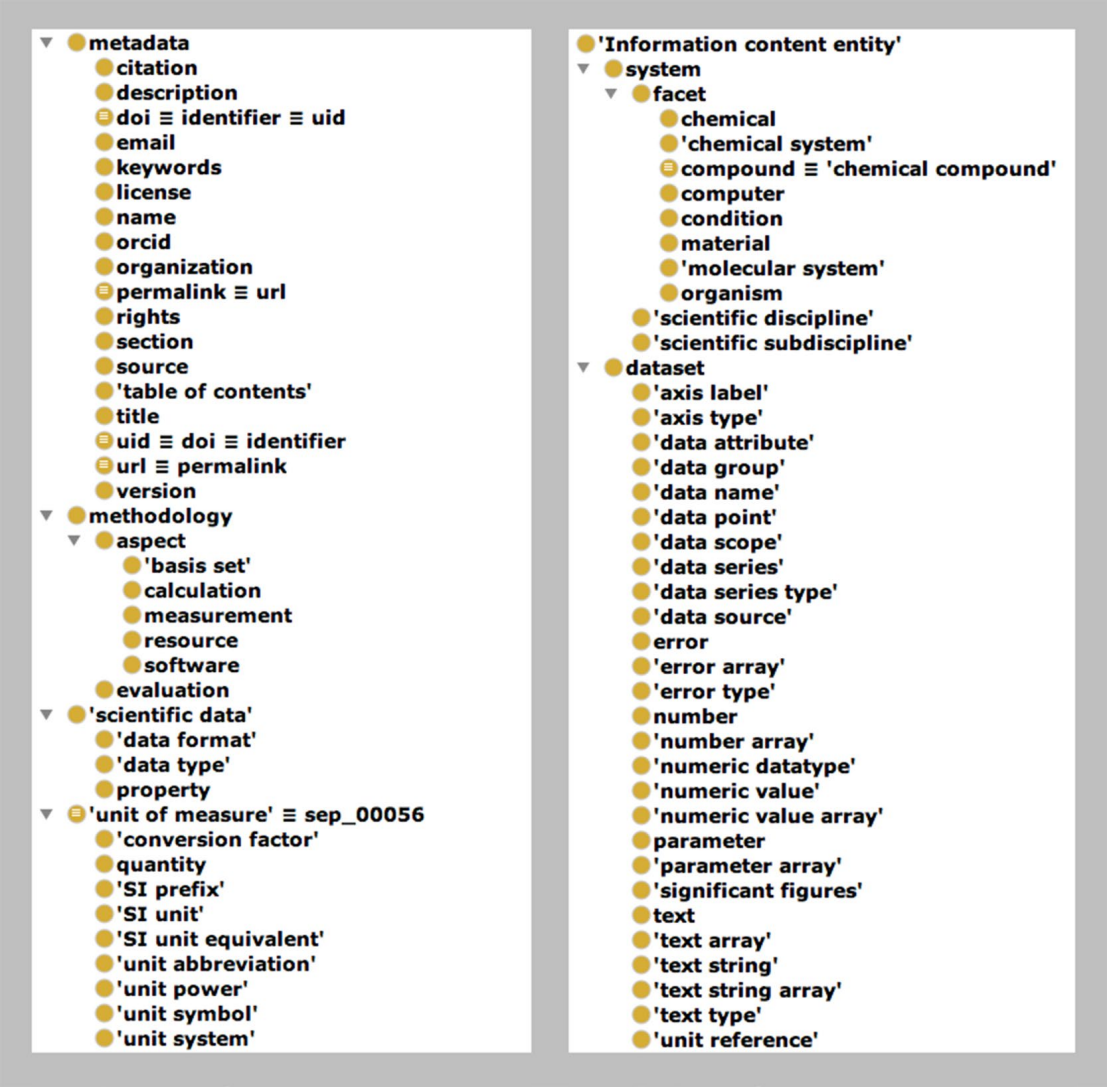
Terms defined in the Scientific Data Model Ontology (SDMO)

**Fig. 11:**
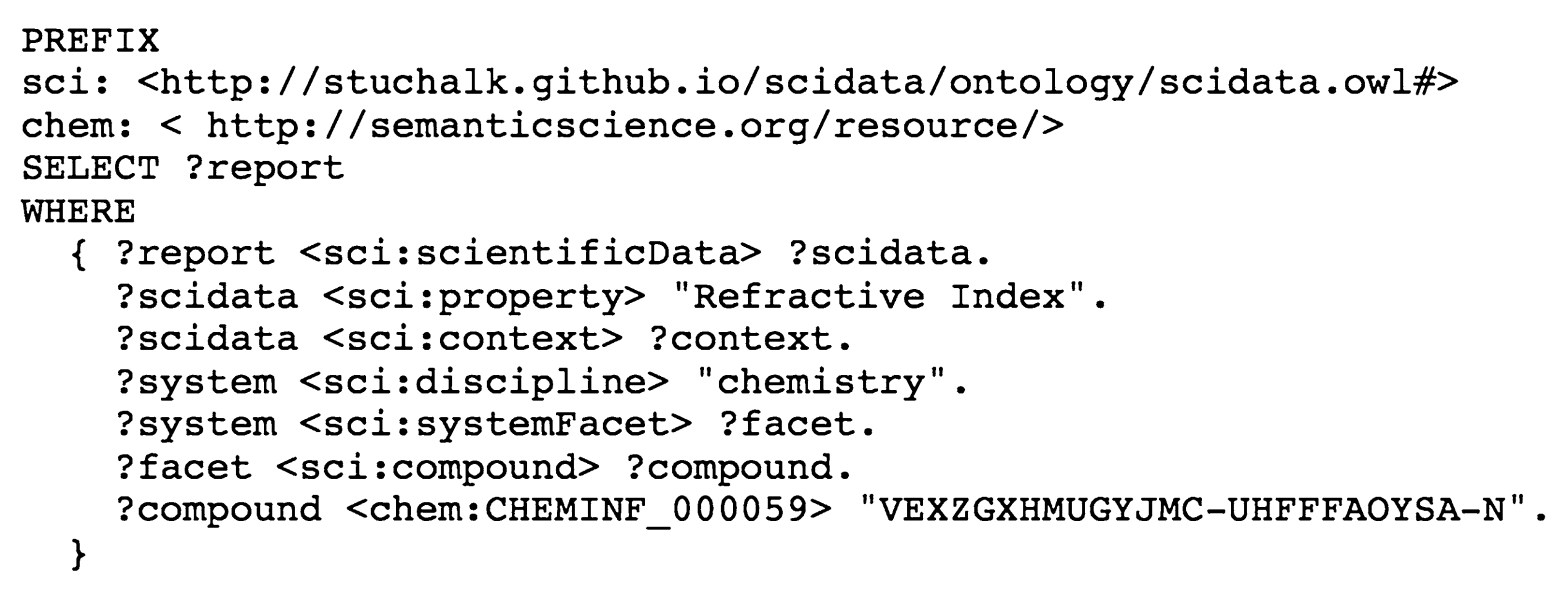
SPARQL query of scientific data

In order to give semantic meaning to the framework created by the SciData scientific data model an associated scientific data model ontology (SDMO) has been developed [54]. Each of the metadata elements that are specific to the framework is included in the ontology (over 60) along with reproduction of common metadata terminology (e.g. from Dublin Core [55]). Terms have been grouped into classes (see Fig. 10): metadata, context, dataset, methodology, scientific data, and unit of measure. The semantic annotation of the framework provides the structure that allows SPARQL [6] searches to be constructed that can mine data from multiple sources. Figure 11 shows an example SPARQL query to find all scientific data reports where a refractive index is reported using hydrochloric acid (via InChI Key) in the area of chemistry.

### Encoding data in the data model

A full discussion of the use of JSON-LD to encode all of the metadata terms described above is beyond the scope of this paper, however, readers interested in viewing/using JSON-LD to explore this approach can go to the project website [1] where the full set of context files can be accessed along with example data documents. In addition, taking example files [56] and loading them into the JSON-LD playground [10] allows readers to see the RDF generated from JSON-LD encoded data.

**Fig. 12:**
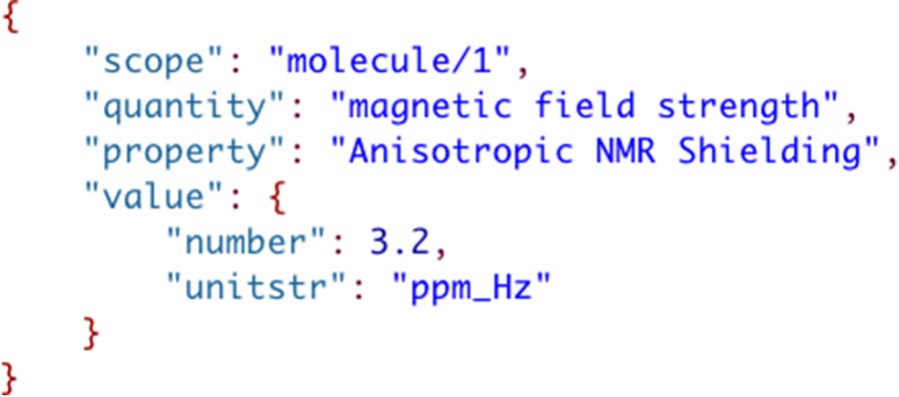
Metadata for calculated parameter value

To illustrate the use of JSON-LD to represent scientific data and what it means consider the JSON text below for a ‘parameter’ (Fig. 12). The JSON object represented in the figure is a collection of metadata strings and an embedded JSON object that represents the value of the parameter. Although a human can relatively easily understand the meaning of information presented, a computer sees the structure as strings and a numeric value. In order to add the meaning to this information so that a computer can represent it a JSON-LD context [57] needs to be included to reference the meanings of each of the JSON name-value pairs.

**Fig. 13:**
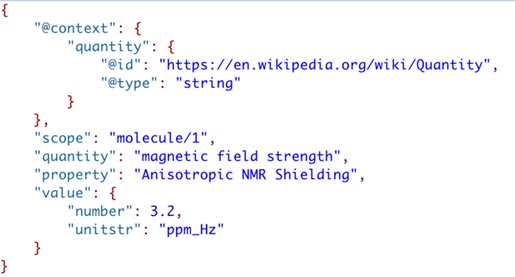
Addition of a JSON-LD context element to the parameter value

**Fig. 14:**
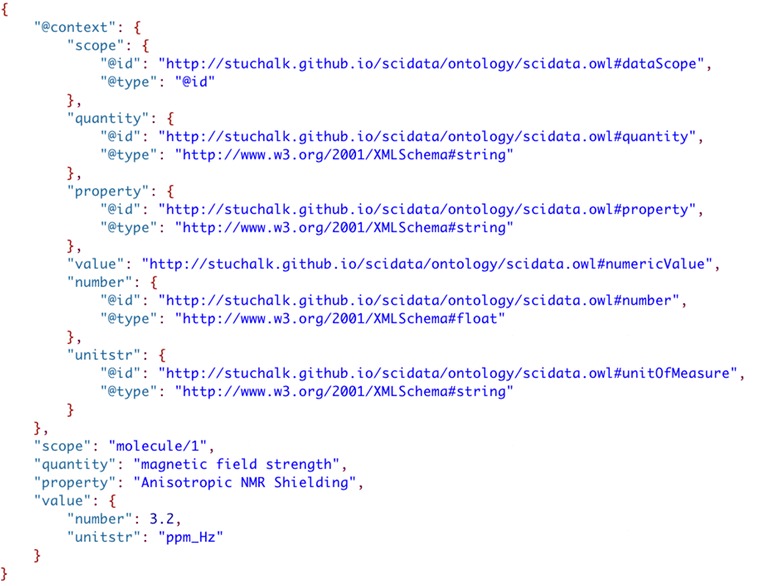
Adding all JSON-LD “term definition” declarations to the context

JSON-LD contexts [57] are indicated in a JSON file by the addition of a “@context” JSON object (see Fig. 13). In this example, the “ontological term definition” for “quantity” is added as a shortcut called “quantity” using a Uniform Resource Identifier (URI) (indicated by “@id”) where a definition of the term is reported, and the value “magnetic field strength” is indicated by “@type” as being of type “string”. Adding term definitions for all name-value pairs gives the JSON-LD file in Fig. 14.

**Fig. 15:**
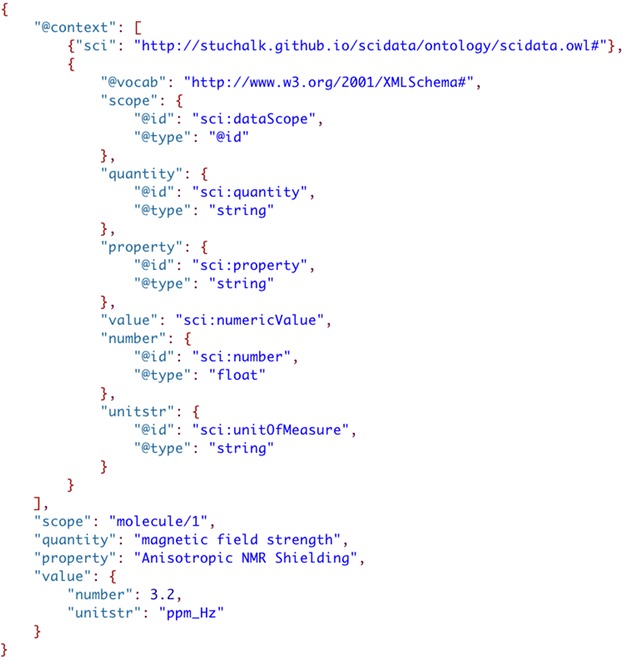
Cleaning up the URI term definitions in the context

It can be seen that providing term definitions for all the elements and including all the full URIs makes the file much larger and complicated. Luckily, there are shortcuts that can be implemented to tidy things up quite a bit, i.e. the inclusion of a namespace abbreviation for the ontology URI (“sci”) and the definition of a “@vocab” assignment to shorten the references to the data types. Figure 15 shows the cleaned up context array.

**Fig. 16:**
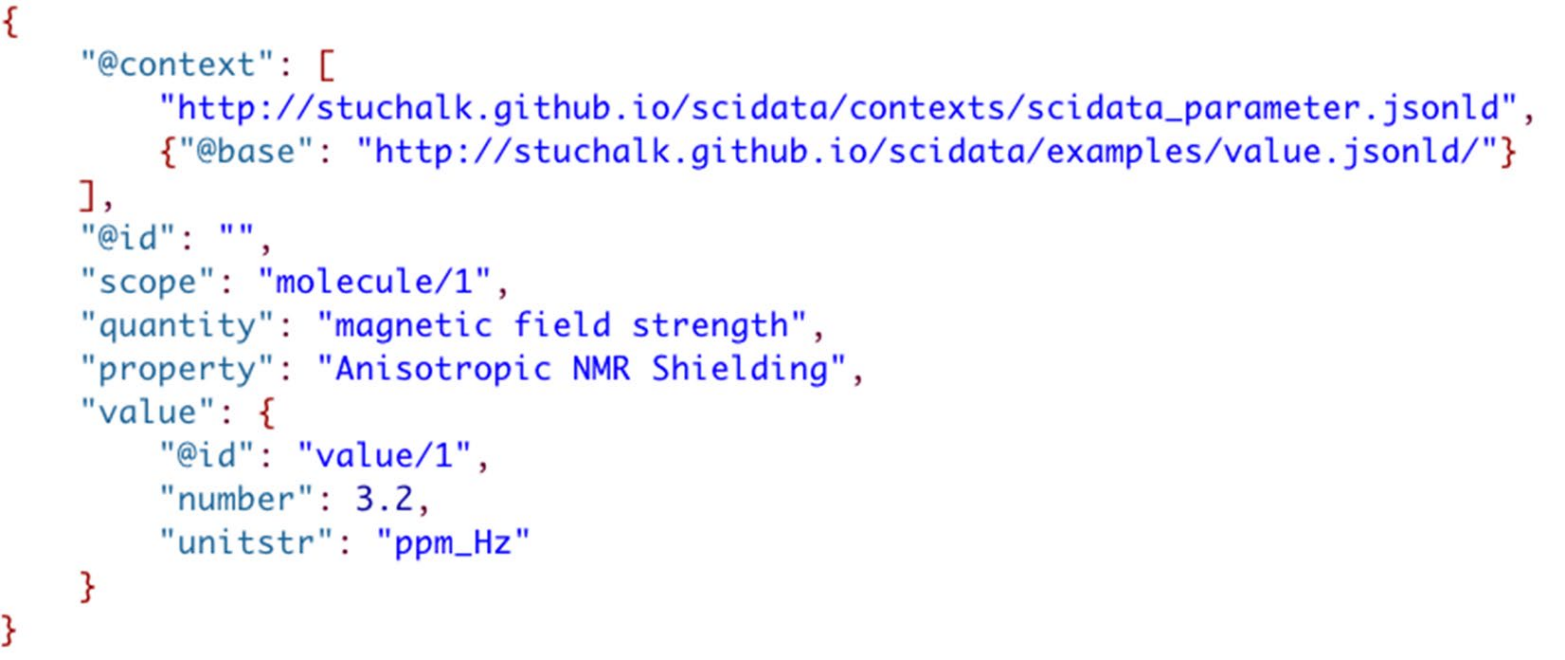
Using an external context file and adding document references to parameter data (“@base” and “@id”)

**Fig. 17:**
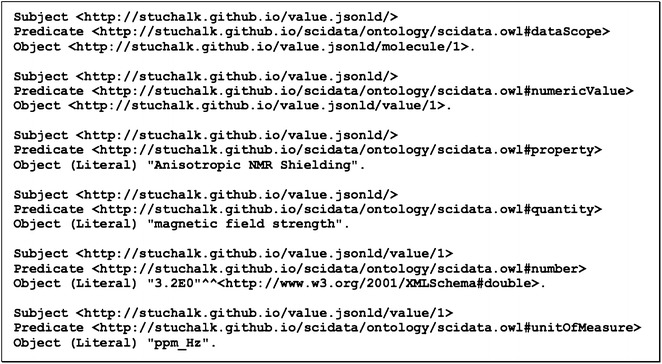
RDF triples corresponding to the linked data in the JSON-LD parameter file in Fig. 10

Finally, to ensure that this context specification can be used across many documents it can be extracted from the data file and saved as a stand-alone context file that is referenced in the parameter file (Fig. 16). Also note in Fig. 10 that an “@id” field is added to the root of each JSON object. This allows (along with the definition of the “@base” attribute) the generation of a unique URI for the parameter and separately its value. Copying and pasting this document into the JSON-LD playground [10] results in the triples shown in Fig. 17.

### Application of the data model

The following portions of four examples show the application of the data model to different data needs. Each of these examples can be found in full on the example page of the project website 1. Additional examples showing the conversion of example data from PubChem, the Dortmund Data Bank, and a CIF file are also included on this page along with XML Stylesheet Language Transformation (XSLT) [58] files used to convert them.

#### The pH of a solution

**Fig. 18:**
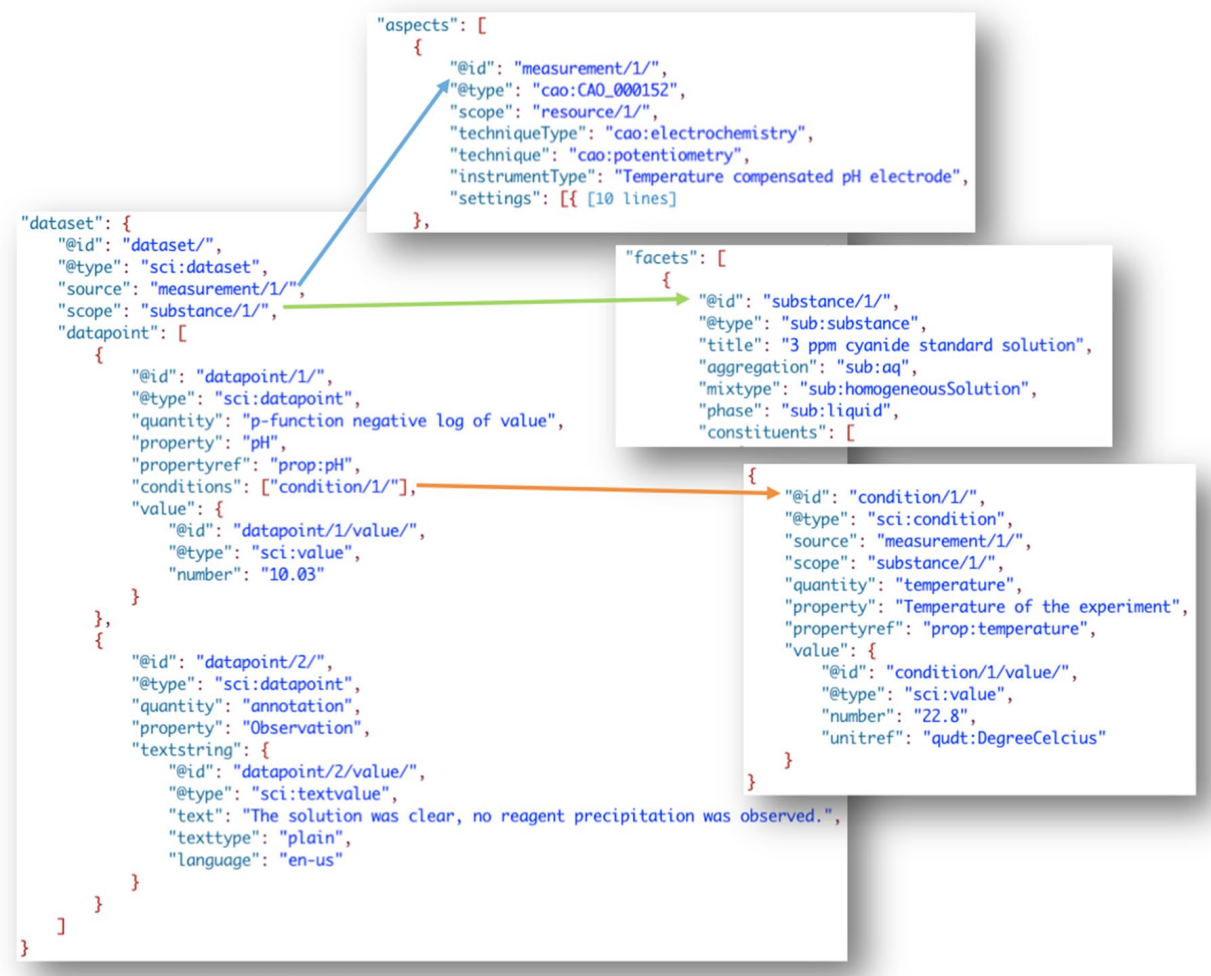
SciData JSON-LD representation of numeric and textual data points

This is an example of the most generic type of data—that of individual data points. In of itself a data point is the reporting of a numeric (or textual) value with or without a unit. In this case pH is measured along with an observation of a solution (Fig. 18). Included with the data are references to other parts of the file that contain the data about the measurement, substance, and condition under which the measurement was made (see [56] for complete file).

#### A measured property extracted from the literature

**Fig. 19:**
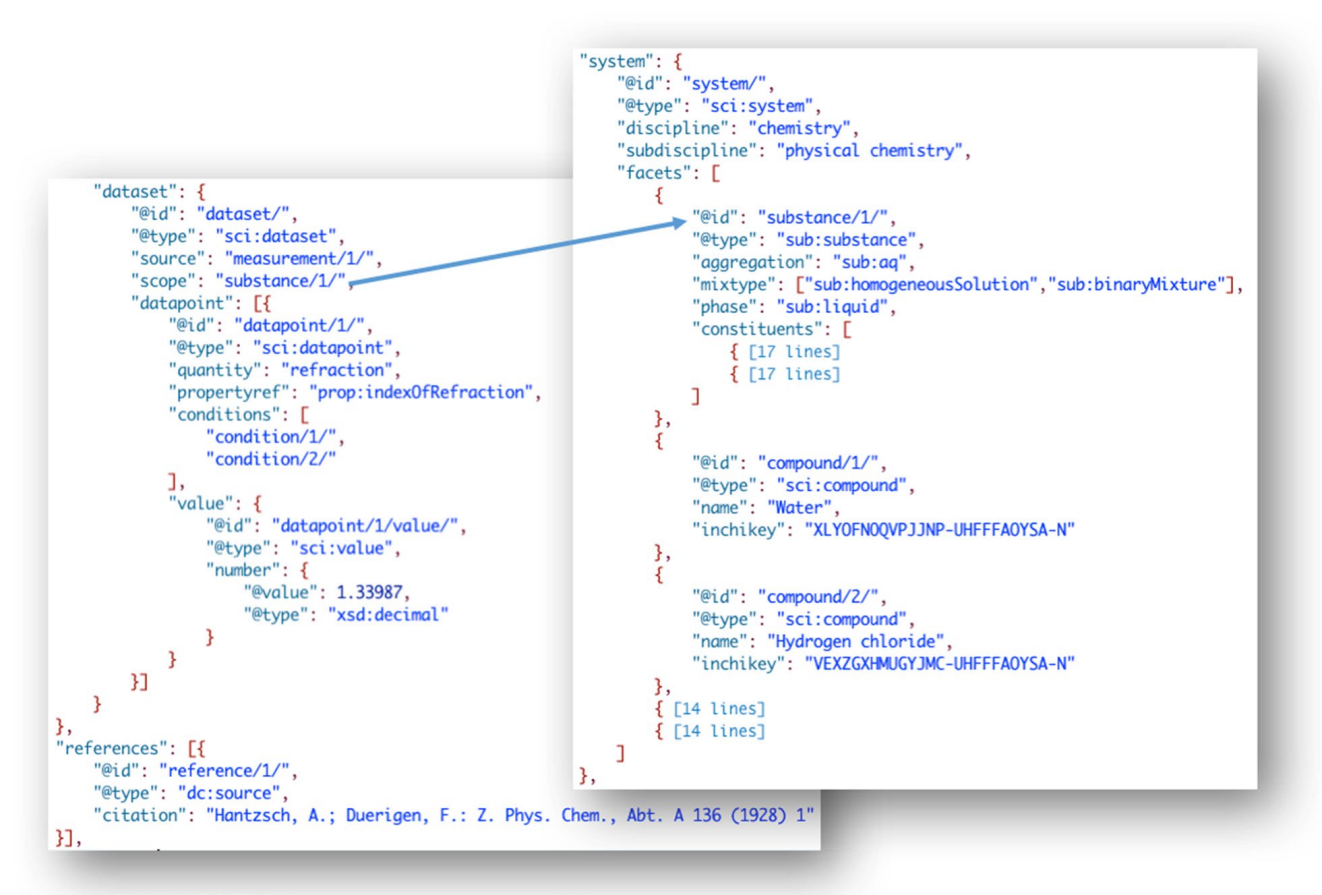
SciData JSON-LD representation of the refractive index of hydrochloric acid from a research paper

In order to make use of data reported previously in this linked-data age it is necessary to go to an original article and extract the reported value and its metadata into the data model. Below, in Fig. 19, is the data and original paper reporting the refractive index of a hydrochloric acid solution. Although not shown, the file also contains information about the measurement and conditions equivalent to that shown in Fig. 18 (see [56] for complete file).

#### NMR spectrum of a sample of R-(+)-Limonene

**Fig. 20:**
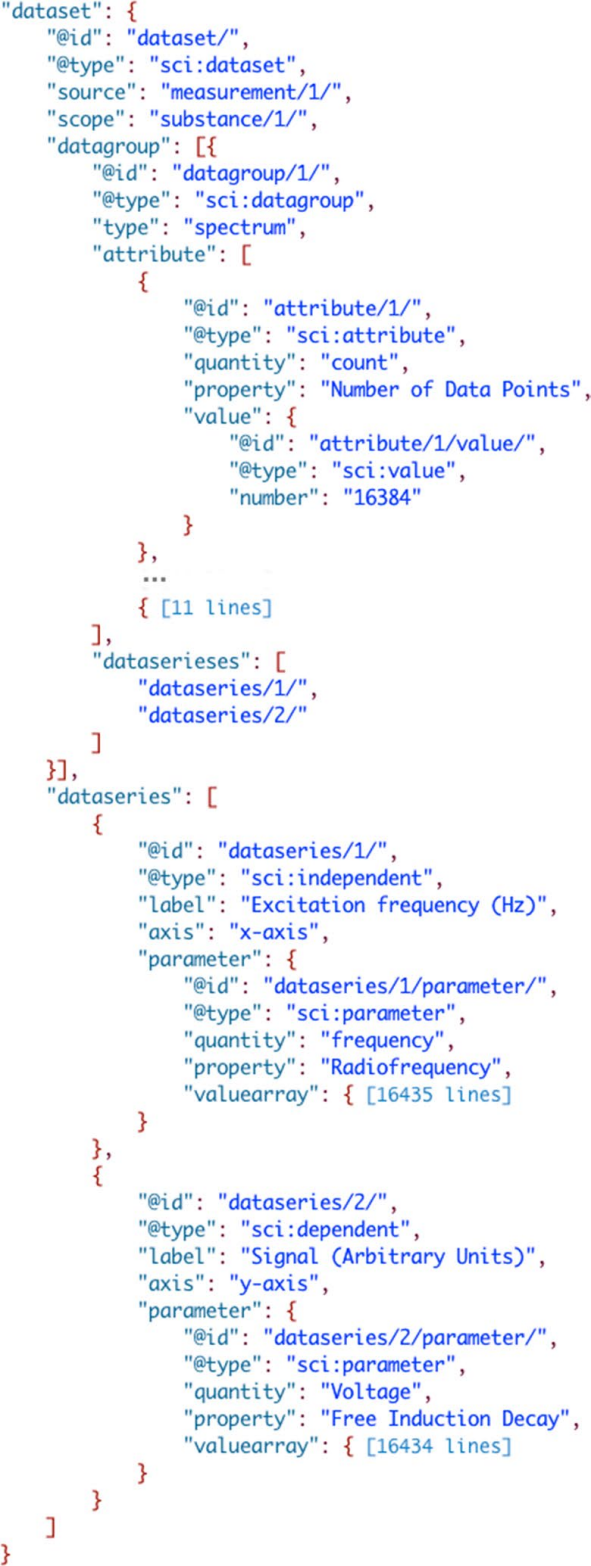
SciData JSON-LD representation of an NMR spectrum of R-(+)-Limonene

The majority of scientific data is measured using analytical instrumentation that produces data as spectra, chromatograms (2D and 3D data), kinetic traces, fiagrams, and many more. The collection and storage of this data can be readily done in ‘dataseries’ but some mechanism is needed to aggregate related ‘dataseries’. This can be done using a generic data group structure where its contents are two ‘dataseries’ plus additional metadata to describe what type of data group it is. Below (Fig. 20) is an example for storing a Nuclear Magnetic Resonance (NMR) spectrum (see [56] for complete file).

#### Computational chemistry calculation of electronic properties of glucose

**Fig. 21:**
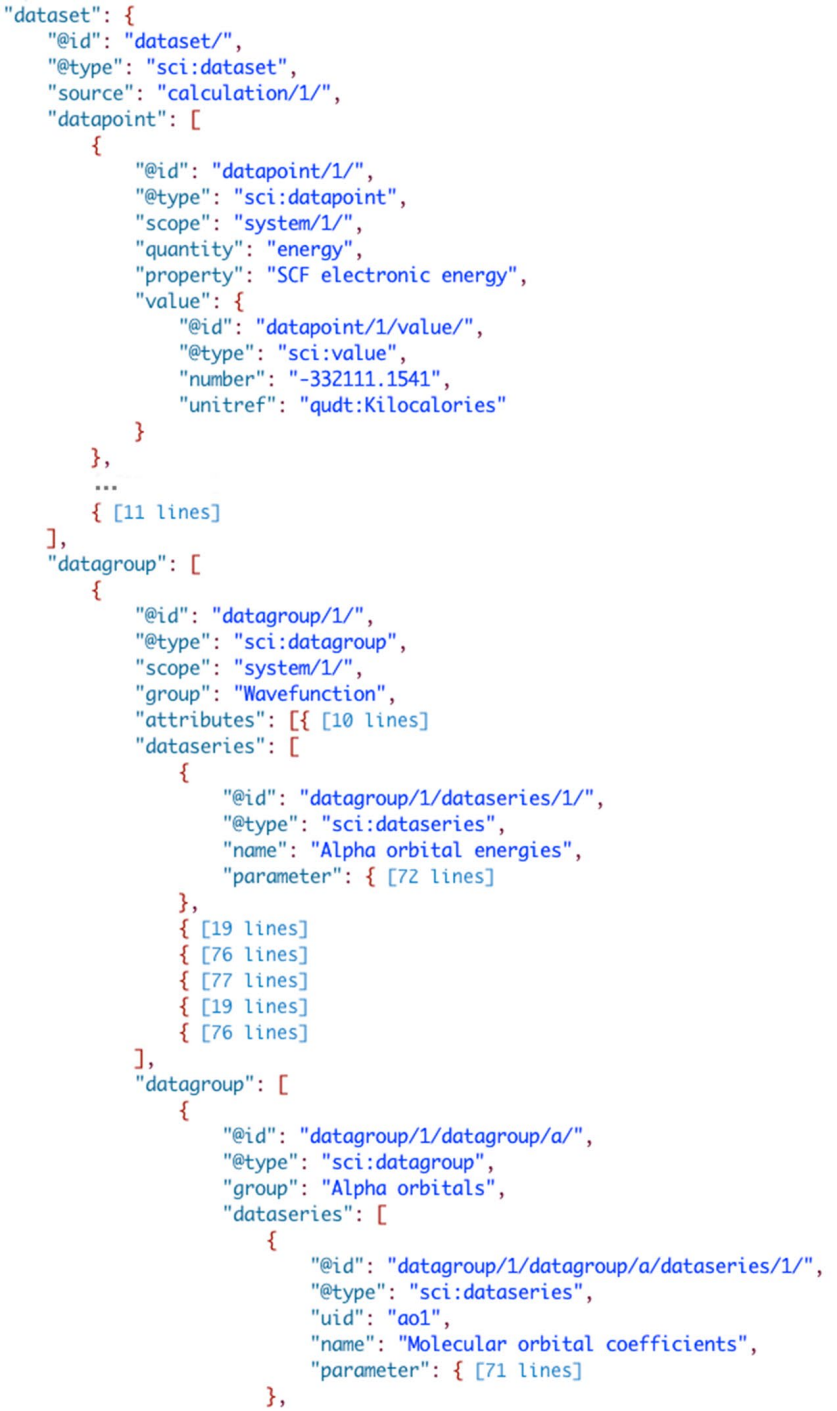
SciData JSON-LD representation (partial) of the results from a glucose SCF calculation

The last example shows how results from computational chemistry calculations can be captured in the SciData format (Fig. 21). A large amount of data is generated from the calculations (the JSON-LD file is over 17,000 lines long) yet the use of nested ‘datagroup’s allows straightforward organization of the spectrum data (see [56] for complete file).

## Conclusion

With the current interest in big data and the movement toward open science there is a need for approaches to allow science data to be made available in an open and easily searchable format. This format needs to be flexible enough to accommodate data from scientific experiments of all kinds and the SciData data model and its implementation in JSON-LD fits that need. Assuming that this, or another framework, is accepted by the scientific community to collect, store, and disseminate semantically annotated scientific data, we can move to the next phase of tool development and data integration to move us toward the utopia of open, accessible and reliable semantically annotated scientific data.

## Abbreviations

CCDC: Cambridge Crystallographic Data Centre
JSON: JavaScript Object Notation
JSON-LD: JavaScript Object Notation for Linked Data
NMR: nuclear magnetic resonance
NoSQL: not only SQL
OWL: Web Ontology Language
REST: Representational State Transfer
RDF: Resource Description Framework
SDM: scientific data model
SDMO: scientific data model ontology
SPARQL: SPARQL Protocol and RDF Query Language
SQL: Structured Query Language
URI: Uniform Resource Identifier
W3C: World Wide Web Consortium
XML: Extensible Markup Language
XSLT: XML Stylesheet Language Transformation
